# A Dual GLP-1/GIP Receptor Agonist Does Not Antagonize Glucagon at Its Receptor but May Act as a Biased Agonist at the GLP-1 Receptor

**DOI:** 10.3390/ijms20143532

**Published:** 2019-07-19

**Authors:** Noura Al-Zamel, Suleiman Al-Sabah, Yunus Luqmani, Lobna Adi, Siby Chacko, Tom Dario Schneider, Cornelius Krasel

**Affiliations:** 1 Department of Pharmacology & Toxicology, Faculty of Medicine, Kuwait University, PO Box 24923, 13110 Safat, Kuwait; 2 Department of Pharmaceutical Chemistry, Faculty of Pharmacy, Kuwait University, PO Box 24923, 13110 Safat, Kuwait; 3 Institute of Forensic Medicine, Department of Forensic Pharmacology and Toxicology, University of Zurich, 190/52 CH-8057 Zurich, Switzerland; 4 School of Pharmacy, Institute for Pharmacology and Toxicology, The Philipps University of Marburg, Karl-von-Frisch-Straße, 135033 Marburg, Germany

**Keywords:** GLP-1, GIP, glucagon, receptor, arrestin

## Abstract

Glucagon-like peptide-1 (GLP-1) and glucose-dependent insulinotropic polypeptide (GIP) are important regulators of metabolism, making their receptors (GLP-1R and GIPR) attractive targets in the treatment of type 2 diabetes mellitus (T2DM). GLP-1R agonists are used clinically to treat T2DM but the use of GIPR agonists remains controversial. Recent studies suggest that simultaneous activation of GLP-1R and GIPR with a single peptide provides superior glycemic control with fewer adverse effects than activation of GLP-1R alone. We investigated the signaling properties of a recently reported dual-incretin receptor agonist (P18). GLP-1R, GIPR, and the closely related glucagon receptor (GCGR) were expressed in HEK-293 cells. Activation of adenylate cyclase via Gα_s_ was monitored using a luciferase-linked reporter gene (CRE-Luc) assay. Arrestin recruitment was monitored using a bioluminescence resonance energy transfer (BRET) assay. GLP-1, GIP, and glucagon displayed exquisite selectivity for their receptors in the CRE-Luc assay. P18 activated GLP-1R with similar potency to GLP-1 and GIPR with higher potency than GIP. Interestingly, P18 was less effective than GLP-1 at recruiting arrestin to GLP-1R and was inactive at GCGR. These data suggest that P18 can act as both a dual-incretin receptor agonist, and as a G protein-biased agonist at GLP-1R.

## 1. Introduction

The incretins, glucose-dependent insulinotropic polypeptide (GIP), and glucagon-like peptide-1 (GLP-1) are peptide hormones secreted from the gut in response to feeding and act to lower postprandial glycaemia by potentiating glucose-induced insulin secretion [1,2]. Their receptors (GIPR and GLP-1R, respectively) are expressed on pancreatic β-cells as well as other cell types, conferring various extra-pancreatic actions to these hormones [3]. For example, GLP-1 reduces appetite and may have cardio- and neuroprotective effects, whereas GIP regulates adipose tissue and bone metabolism [4,5]. An impairment of action of the incretin hormones has been identified as an early and specific characteristic of type-2 diabetes mellitus (T2DM) and is likely to be due to a loss of response to the incretin hormones at the pancreatic β-cell [6]. Pharmacological levels of GLP-1 can overcome this resistance and as a result of various GLP-1R agonists have been approved for the treatment of T2DM and obesity [7]. While the same is not true for GIP, co-administration of GIP to diabetic patients resulted in enhancement of the efficacy of GLP-1. This observation has led to the development of a single molecule that can act equally on GIPR and GLP-1R. This dual incretin receptor agonist was more effective at correcting hyperglycemia than selective agonists for each receptor, and in lowering bodyweight in animal models of T2DM and obesity and represents a new strategy for the treatment of these disorders [8].

The receptors for GIP and GLP-1 are members of the Class B (or secretin) family of G protein˗coupled receptors (GPCRs). They share approximately 40% sequence homology and both couple positively to adenylate cyclase through Gα_s_ G proteins, resulting in an increase in intracellular cyclic adenosine monophosphate (cAMP) [9,10,11]. Originally thought of as proteins that regulate the homologous desensitization and internalization of GPCRs, arrestins are now appreciated as adaptor molecules that also allow GPCRs to signal through G protein-independent pathways such as ubiquitin ligases, mitogen-activated protein (MAP) kinases, and Src tyrosine kinases [12,13]. Although several groups have demonstrated that GLP˗1R can recruit arrestin2 and arrestin3 [14,15], both desensitization and internalization appear to be arrestin-independent processes for GLP-1R [16]. Furthermore, knockdown of arrestin2 in cultured pancreatic β-cells resulted in impairment of GLP-1 to stimulate cAMP and insulin production [17]. This suggests that arrestin-recruitment is an integral component of GLP-1 signaling. In contrast, a recently reported G protein biased GLP-1R agonist, with a reduced ability to recruit arrestin compared to other FDA-approved GLP-1R agonists, displayed potent long term glycemic benefits in a mouse model of type 2 diabetes without the commonly associated nausea that limits the dose of this class of treatment [18]. This enhanced insulin release was associated with an ability to hold GLP-1R at the plasma membrane and faster agonist dissociation rates. Unlike GLP-1R, it remains unclear if GIPR can recruit arrestin. Some groups showed no interaction with arrestin [15,19] while others did [20].

**Figure 1:**
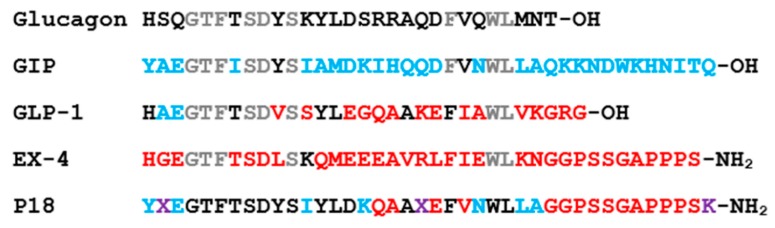
Peptide ligands used in this study. Residues that are derived from those of glucagon are shown in black, from glucose-dependent insulinotropic polypeptide (GIP) in blue and from glucagon-like peptide-1 (GLP-1) and exendin-4 (Ex-4) in red. Residues shown in grey are shared by glucagon, GIP, GLP-1, and Ex-4. Residues shown in purple are unique to P18, X = aminoisobutyric acid. GLP-1 and Ex-4 are C-terminally amidated (Adapted from Finan et al., 2013 [8]).

In the present study, we investigated the selectivity and signaling properties of a dual GLP-1/GIP receptor agonist (Peptide 18 (P18); Figure 1) using HEK-293 cells expressing either human GLP-1R, GIPR or the closely related glucagon receptor (GCGR). cAMP production following receptor activation was monitored using a highly sensitive cAMP-responsive luciferase assay. The ability of P18 to antagonize glucagon at its receptor was also investigated. Arrestin recruitment was measured using a bioluminescence resonance energy transfer (BRET)-based assay to investigate the G protein-independent/arrestin-dependent component of signaling.

## 2. Results

### 2.1. Activity at the GLP-1 Receptor

**Figure 2:**
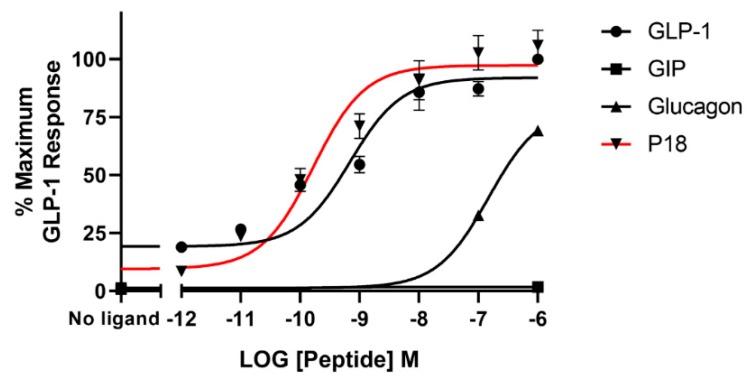
Peptide ligand activity at the GLP-1 receptor expressed in HEK-293 cells. Data represent the mean ± S.E.M. from at least three independent experiments, each performed in triplicates. The counts were normalised to the maximum GLP-1 response.

Using a cAMP-responsive luciferase assay, GLP-1, GIP, glucagon and P18 (peptide sequences shown in Figure 1) were tested for their ability to activate the GLP-1 receptor. Dose–response curves are shown in Figure 2 and the peptide’s *p*EC_50_ and E*_max_* values are shown in Table 1. GLP-1 and P18 exhibited similar potency and E*_max_* values at the GLP-1 receptor. Glucagon activity was only detectable at concentration of 1 µM and GIP activity was not detectable at the concentrations tested. As a result of glucagon’s low potency at GLP-1R, the *p*EC_50_ value for this peptide shown in Table 1 is simply an estimate.

### 2.2. Activity at the GIP Receptor 

**Figure 3:**
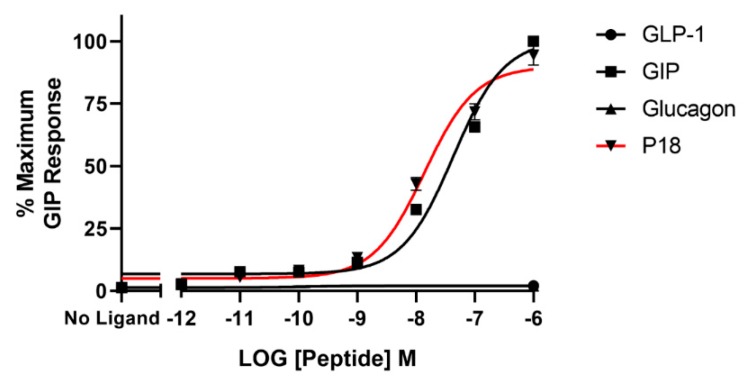
Peptide ligand activity at the GIP receptor expressed in HEK-293 cells. Data represent the mean ± S.E.M. from at least three independent experiments, each performed in triplicates. The counts were normalised to the maximum GIP response.

The same four peptides were tested for their ability to activate the GIP receptor. Dose–response curves are shown in Figure 3 and the peptide’s *p*EC_50_ and E*_max_* values are shown in Table 1. P18 was almost an order of magnitude more potent than GIP at the GIP receptor but both peptides showed similar E*_max_*. Neither glucagon nor GLP-1 displayed any detectable activity at the GIP receptor at the concentrations tested.

### 2.3. Activity at the Glucagon Receptor

**Figure 4:**
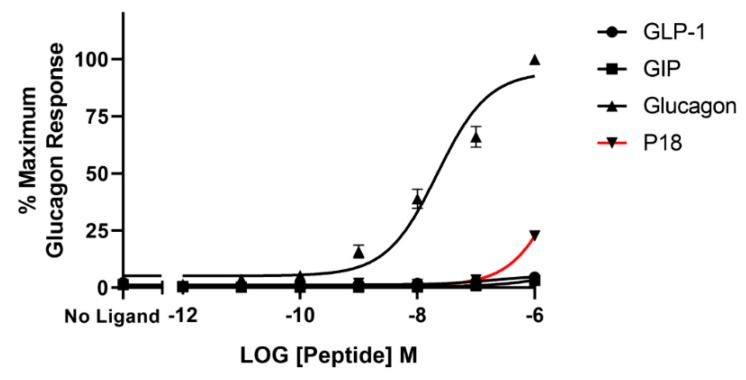
Peptide ligand activity at the glucagon receptor expressed in HEK-293 cells. Data represent the mean ± S.E.M. from at least three independent experiments, each performed in triplicates. The counts were normalised to the maximum glucagon response.

**Figure 5:**
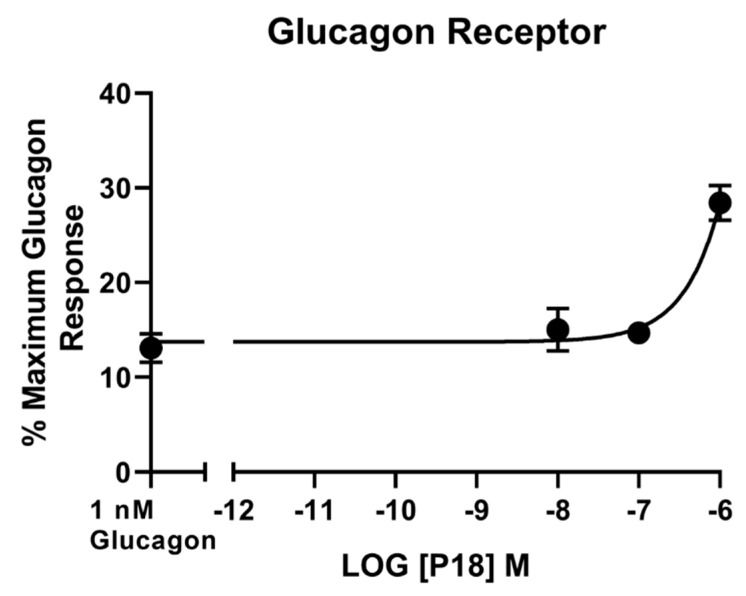
Increasing concentrations of P18 did not inhibit the response to 1 nM glucagon at the glucagon receptor expressed in HEK-293 cells. P18 exerted no effect on stimulation with glucagon, except at concentrations of 1 µM, for which a small but significant (*p* < 0.05) increase in activity compared to 1 nM glucagon alone was observed. Data represent the mean ± S.E.M. from at least three independent experiments, each performed in triplicates. The counts were normalised to the maximum glucagon response.

Glucagon was the only peptide tested that acted as a full agonist at the glucagon receptor. Dose–response curves are shown in Figure 4 and the peptide’s *p*EC_50_ and E*_max_* values are shown in Table 1. GLP-1 and GIP activity at the glucagon receptor could not be detected at concentrations up to 1 µM and P18 activity could only be detected at concentrations of 1 µM. As a result of P18′s low potency at GCGR, the *p*EC_50_ value for this peptide shown in Table 1 is simply an estimate. Increasing concentrations of P18 were unable to antagonize the activity of 1 nM glucagon at the glucagon receptor (Figure 5). Interestingly, a small but significant (*p* < 0.05) increase in activity was observed when the concentration of P18 reached 1 µM.

### 2.4. Bioluminescence Resonance Energy Transfer (BRET) Assays

**Figure 6:**
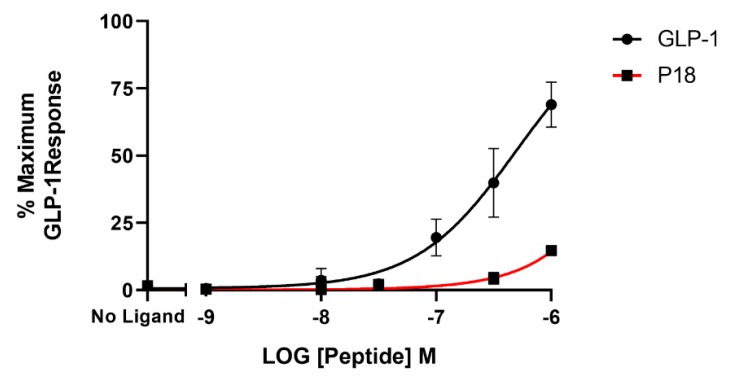
Arrestin recruitment (represented by the BRET ratio) by GLP-1 or P18 to GLP-1R-SYFP2 expressed in Flip-In HEK-293 cells stably expressing Arr3-Nluc. Data represent the mean ± S.E.M. from at least three independent experiments, each performed in triplicates. The counts were normalised to the maximum GLP-1 response.

**Figure 7:**
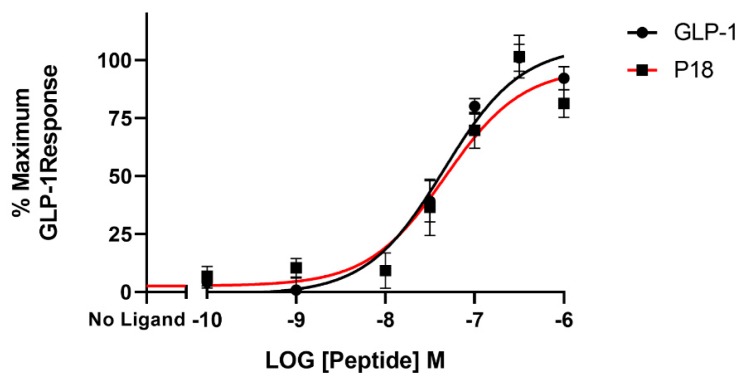
Gα_S_ recruitment (represented by the BRET ratio) by GLP-1 or P18 to GLP-1R-Nluc expressed in Flip-In HEK-293 cells. Data represent the mean ± S.E.M. from at least three independent experiments, each performed in triplicates. The counts were normalised to the maximum GLP-1 response.

**Figure 8:**
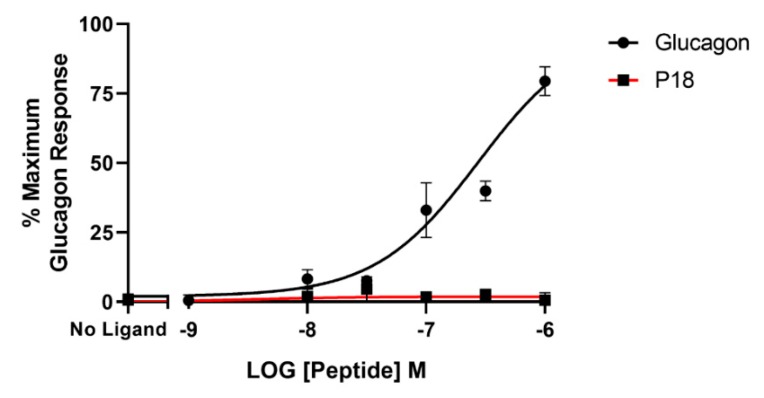
Arrestin recruitment (represented by the BRET ratio) by glucagon or P18 to GCGR-SYFP2 expressed in Flip-In HEK-293 cells stably expressing Arr3-Nluc. Data represent the mean ± S.E.M. from at least three independent experiments, each performed in triplicates. The counts were normalised to the maximum glucagon response.

BRET assays were used to monitor both arrestin3 and Gα_s_ recruitment to the GLP-1 receptor. Treatment with GLP-1 dose-dependently increased arrestin recruitment to the GLP-1 with a mean *p*EC_50_ of 6.4 ± 0.2 S.E.M for 3 independent experiments, whereas P18 stimulation of arrestin recruitment to the GLP-1 receptor was only detectable at concentrations of 1 µM. Dose-response curves are shown in Figure 6. In contrast, there was no significant difference in the potency of GLP-1 or P18 to stimulate Gα_s_ recruitment to the GLP-1 receptor (Figure 7). Their respective mean *p*EC_50_ values of 7.4 ± 0.1 S.E.M. and 7.4 ± 0.1 S.E.M for 4 independent experiments were identical. Arrestin recruitment to the GIP receptor was not detectable using the BRET assay (data not shown). Glucagon stimulated arrestin recruitment to the glucagon receptor with a similar potency (*p*EC_50_ of 6.8 ± 0.3 S.E.M for 3 independent experiments) as GLP-1 stimulated arrestin recruitment to its receptor. P18 did not stimulate arrestin recruitment to the glucagon receptor (Figure 8).

## 3. Discussion

Several GLP-1R agonists (e.g., exenatide, liraglutide, and semaglutide) are currently used clinically to treat both T2DM and obesity [21]. While they reduce mortality, improve glycemic control, reduce body weight and improve cardiovascular risk factors, the use of higher doses is often limited by adverse effects such as nausea and vomiting [22,23]. There is therefore the potential to improve upon the current class of GLP-1R agonists. Targeting GIPR to treat T2DM and obesity has a somewhat contradictory history. Unlike GLP-1, pharmacological doses of exogenous GIP were not found to be insulinotropic in patients with type 2 diabetes [24]. Interest in GIPR antagonists developed after studies where GIPR knockout mice were shown to be resistant to diet-induced obesity [25]. This led to the development of a derivative of GIP with a glutamic acid to proline substitution at position three. Pro3GIP, initially reported to be a GIPR antagonist, was shown to be protective against diabetes and obesity in rodent models [26,27]. However, subsequent studies have since demonstrated that Pro3GIP is, in fact, a low potency GIPR agonist [28,29]. Furthermore, GIP overexpressing mice exhibit reduced diet-induced obesity and improved glucose homeostasis [30]. There is also the possibility that resistance to GIP observed in patients with type 2 diabetes can be overcome by lowering circulating levels of glucose [31]. Taken together, this suggests that GIP may have some utility in the treatment of type 2 diabetes when combined with GLP-1. Indeed, dual GLP-1/GIP receptor agonists have been developed that have greater efficacy in terms of weight loss and glycemic control than either peptide alone. [8,32].

In the present study, we investigated the activity of a previously reported dual GLP-1/GIP receptor agonist at GLP-1R, GIPR and the closely related GCGR expressed in HEK-293 cells using a luciferase-coupled cAMP response element reporter gene assay [8]. In agreement with previous studies, GLP-1, GIP, and glucagon were highly selective for their respective receptors [28,33]. Neither GLP-1 nor GIP displayed any activity at receptors other than their own at the concentrations used in this study. Glucagon only displayed detectable activity at GLP-1R at concentrations of 1 µM. The dual GLP-1/GIP receptor agonist, P18, activated GLP-1R with similar potency to that of the native peptide and achieved a similar E_max_. In contrast, Finan et al., 2013 [8] have reported that P18 was 177% more potent than GLP-1 at GLP-1R. This discrepancy is most likely because these authors used EC_50_ (nM) values to calculate relative activity whereas we used *p*EC_50_ values. Finan et al. report an EC50 of 0.028 nM for GLP-1 and 0.016 nM for P18, which equates to *p*EC_50_ values of 10.55 and 10.79 respectively. This small difference in potency is of a similar scale to our observation. However, P18 was significantly (*p* < 0.05) more potent than GIP at GIPR and only had detectable activity at GCGR at concentrations of 1 µM, demonstrating that in terms of cAMP production P18 is a dual incretin receptor agonist, which is in agreement with Finan et al., 2013 [8]. Although our observed values for potency are lower than those reported by Finan et al., 2013 [8] the rank orders of potency are the same. This is not surprising as different assays are likely to produce different values for potency due to different levels of receptor expression for example.

We also conducted experiments to determine whether P18 could act as an antagonist at the GCGR as blocking the action of glucagon has been proposed as a novel strategy in the treatment of various forms of diabetes [34]. Increasing concentrations of P18 had no inhibitory effect on the response to 1 nM glucagon at GCGR. However, a small but significant (*p* < 0.05) increase in activity was observed when concentrations of P18 reached 1 µM which is perhaps not surprising as this peptide was capable of activating GCGR to a small degree at this concentration. Due to their similarity in sequence it is possible that P18 has affinity, albeit low, for GCGR. These data suggest that the beneficial effects of P18 are mediated by activation of both GLP-1R and GIPR and not by either GCGR agonism or antagonism.

As it is now appreciated that GPCRs can signal through both G protein-dependent and G protein-independent pathways we investigated the ability of P18 to recruit arrestin to GLP-1R, GIPR, and GCGR using a BRET-based assay. GLP-1 stimulated arrestin recruitment to GLP-1R in a dose dependent manner; in agreement with several previous studies [14,15]. In contrast however, P18 was unable to stimulate any observable arrestin recruitment to GLP-1R except at a concentration of 1 µM. Furthermore, the BRET ratio (a readout for arrestin recruitment) resulting from stimulation with this concentration of P18 was significantly less (*p* < 0.0001) than that stimulated by the same concentration of GLP-1. These data would suggest that P18 is a G protein-biased agonist. However, the Cre-Luc assay we employed to measure G protein activation is a highly amplified system, several steps downstream of the receptor, whereas the BRET-based arrestin recruitment assay directly measures the interaction between the receptor and the arrestin molecule. To confirm that P18 is in fact a G protein-biased agonist at GLP-1R we utilized a similar BRET-based assay to measure Gα_s_ recruitment to GLP-1R. In this assay, GLP-1 and P18 stimulated Gα_s_ to GLP-1R with almost equal potency and exhibited similar E_max_. This confirms that P18 is a G protein-biased agonist at GLP-1R relative to GLP-1, although it should be noted that in the arrestin recruitment assay the receptor was transiently expressed whereas in the Gα_s_ recruitment assay the receptor was stably expressed. However, the two BRET assays are more comparable than the reporter gene assays as they report events closer to receptor activation and were incubated with agonist for the same length of time. Unlike P18, glucagon also stimulated arrestin recruitment to GCGR. We were unable to observe any GIP or P18 stimulated arrestin recruitment to GIPR. This observation is in agreement with previous studies showing that GIPR does not recruit arrestin effectively [15,19] but contrary to some others [20]. 

The concept of biased agonism (also referred to as ligand-directed signaling or functional selectivity) at GPCRs has received much attention recently as it may lead to the development of novel treatments with greater efficacy or fewer adverse effects than current therapies [35,36]. For example, the clinically desirable effects may be mediated by a G protein-dependent pathway and the adverse effects mediated by an arrestin independent pathway or vice versa. A balanced or unbiased agonist would activate both pathways whereas a biased agonist would favour one pathway over the other. G protein-biased ligands for GLP-1R have been shown to enhance insulin secretion in mouse models of type 2 diabetes without producing nausea, a common adverse effect of GLP-1R agonists, thus allowing higher doses to be used. [18]. These findings are initially surprising, as arrestin recruitment to GLP-1R has also been shown to be a key component to the GLP-1-mediated enhancement of insulin secretion in pancreatic β-cells [16,17]. It has been suggested that this apparent contradiction could be explained by differences in acute and chronic treatments, where less receptor desensitization is observed when using a G protein biased ligand over the long term when compared to a more balanced agonist. Jones et al., 2018 have reported that substitution of the first amino acid residue of the GLP-1R agonist EX-4 from histidine to phenylalanine results in a G protein biased agonist [18]. Interestingly, P18 shares the same first amino acid residue as GIP, which some have reported does not recruit arrestin to its receptor [15,19].

The present study may be extended by ligand binding studies. It would be interesting to correlate the affinities of the various peptides tested for GLP-1R, GIPR, and GCGR with their ability to activate different pathways. In addition, other signaling pathways could be measured, such as Gα_q_ mediated signaling and further downstream molecules such as ERK. Such data could provide useful mechanistic insight and aid in the design of improved novel therapies.

## 4. Materials and Methods

### 4.1. Materials

All peptide ligands (see Figure 1 for sequences) were purchased from Bachem (Bubendorf, Switzerland), with the exception of P18, which was custom synthesised by Pepceuticals Ltd. (Enderby, UK). Cell culture reagents were purchased from Gibco-Invitrogen (Paisley, UK) and Sigma-Aldrich (Poole, UK). General chemicals were purchased from Sigma-Aldrich.

### 4.2. Construction of cDNA

cDNA encoding wild-type human GLP-1R and GIPR constructs have been previously described [28]. cDNA encoding the human glucagon receptor was a gift from Rasmus Jorgensen (Novo Nordisk, Denmark). GLP-1R, GIPR and the glucagon receptor were labelled at the C-terminus with super yellow fluorescent protein 2 (SYFP2) [37] and generated by amplifying the open reading frame of human GLP-1R, GIPR and GCGR with primers which added a HindIII restriction site ahead of the start codon and replaced the stop codon with an XbaI site. The start codon of SYFP2 was replaced by PCR with an XbaI site, and a NotI site was inserted behind the stop codon of SYFP2. The resulting fusion of receptor-SYFP2 was cloned in pcDNA3. 

To construct Arr3-NanoLuciferase (NLuc), PCR was used to insert an XbaI restriction site in front of the NLuc ORF (a kind gift of Nevin Lambert, Augusta University, GA, USA) and a NotI restriction site behind it. These restriction sites were then used to replace the ORF for ECFP in Arr3-CFP [38] with that of NLuc. GLP-1R, GIPR and GCGR labelled at the C-terminus with Nluc were generated by replacing the ORF of Arr3 with that of the receptor using HindIII and XbaI restriction sites. Several constructs were subsequently cloned into pcDNA5-FRT (Invitrogen) in order to generate a stable isogenic cell line.

NES-Venus-Gα_s_ [39] was a gift from Mohammed Ayoub (United Arab Emirates University, Al Ain, United Arab Emirates).

All constructs were verified through Sanger sequencing.

### 4.3. Cell Culture and Transfection of Cells

HEK-293 and Flip-In HEK-293 cells (Invitrogen) were cultured in Dulbecco’s modified Eagle’s media supplemented with 10% foetal calf serum, 100 U/mL penicillin and 100 µg/mL streptomycin. Cells were maintained at 37 °C in a humidified environment containing 5% CO_2_. HEK-293 cells were transiently transfected using Effectene (Qiagen, Hilden, Germany), following the manufacturer’s protocol. In order to generate stable cell lines Flip-In HEK-293 cells were transfected with the pcDNA5.FRT vector and pOG44 using Effectene. Stable isogenic clones were selected by the addition of hygromycin (100 µg/mL).

### 4.4. Luciferase Assay

GLP-1, GIP, and glucagon receptor activation were assessed using a luciferase reporter gene assay, as described previously [40]. Briefly, HEK-293 cells were transiently transfected with cDNA encoding either the GLP-1, GIP or glucagon receptor and a reporter gene construct consisting of a cAMP-response element fused to a reporter gene encoding firefly luciferase (Cre-luc) using Effectene (Qiagen, Hilden, Germany), according to the manufacturer’s instructions. Twenty-four hours after transfection, the cells were seeded into white 96-well plates (Thermo Scientific, Roskilde, Denmark). Twenty-four hours later, the cells were incubated for 3 h in medium containing peptide ligand and then lysed. Luciferase activity was quantified using LucLite reagent (PerkinElmer Life and Analytic Sciences, Wellesley, MA, USA).

### 4.5. Bioluminescence Resonance Energy Transfer (BRET) Assays

For arrestin recruitment assays, FLIP-IN HEK-393 cells stably expressing Arr3-NLuc were transiently transfected with SYFP2-labelled receptor as previously described. For Gα_s_ recruitment assays, FLIP-IN HEK-393 cells stably expressing either the GLP-1R-NLuc or GCGR-NLuc were transiently transfected with NES-Venus-Gα_s_. 48 h post-transfection cells were detached and washed with Hank’s Balance Salt Solution (HBSS). Cells were re-suspended in HBBS and plated on to white 96-well plates (PerkinElmer) in suspension at a density of 180,000 cells/well. Cells were incubated with agonist for 15 min and BRET measurements were taken using a Victor X4 (PerkinElmer) plate reader immediately after the addition of coelenterzine *h* (final conc. 5 µM). NLuc emission was measured through a 460/40 nm filter and the resulting SYFP2 emission was read through a 535/25 nm filter.

### 4.6. Data Analysis

Dose-response curves represent the mean ± S.E.M from at least three independent experiments, each performed in triplicates. The counts were normalised to either the maximum GLP-1, GIP or glucagon response, depending on the receptor being investigated for each data set. The dose-response data were fitted to a sigmoidal curve using nonlinear regression, and the EC_50_ values calculated with the aid of GraphPad 8.0 (GraphPad Dan Diego, CA, USA). The values in Table 1 represent the mean ± S.E.M calculated from the *p*EC_50_ (−logEC_50_) values from at least three independent experiments. Statistical analysis of significance was calculated with GraphPad 8.0 using a two˗tailed, unpaired Student’s *t*-test. 

## 5. Conclusions

The data presented show that P18 is indeed a dual GLP-1/GIP receptor agonist, at least in terms of G protein activation. P18 has no action at the glucagon receptor, indicating that the therapeutic effects of this peptide are not mediated through this receptor. Interestingly, P18 was relatively poor at stimulating arrestin recruitment to GLP-1R compared to GLP-1. This suggests, that as well as dual activation of GLP-1R and GIPR, part of the therapeutic advantage of P18 may be due to its G protein bias at GLP-1R. 

## Figures and Tables

**Table 1:** Activation of GLP-1, GIP and glucagon receptors by peptide ligands.

	GLP-1 Receptor		GIP Receptor		Glucagon Receptor	
	*p*EC_50_	E*_max_* (% GLP-1)	*p*EC_50_	E*_max_*(% GIP)	*p*EC_50_	E*_max_*(% Glucagon)
GLP-1	9.4 ± 0.14	100	ND	ND	ND	ND
GIP	ND	ND	7.1 ± 0.11	100	ND	ND
Glucagon	6.4 ± 0.1 a	62.8 ± 7.4 a	ND	ND	7.5 ± 0.28	100
P18	9.7 ± 0.2	106 ± 10.7	7.8 ± 0.03 b	94.4 ± 4.09	4.9 ± 0.50 c	22.6 ± 1.96 d

The mean ± S.E.M shown are from at least 3 independent experiments ND; no detectable activity at 1 µM. E*_max_* values indicate the maximum luciferase activity as a percentage of either GLP-1, GIP or glucagon activity. *p*EC_50_ refers to −logEC_50_/M. a *p* < 0.01 significantly different from GLP-1 at the GLP-1 receptor, b *p* < 0.05 significantly different from GIP at the GIP receptor, c *p* < 0.005, d *p* < 0.0001 significantly different from glucagon at the glucagon receptor.
