# Correction: Al-Zamel, N., *et al.* A Dual GLP-1/GIP Receptor agonist Does Not Antagonize Glucagon at Its Receptor but May Act as a Biased agonist at the GLP-1 Receptor. *Int. J. Mol. Sci.* 2019, *20*, 3532

**DOI:** 10.3390/ijms21093357

**Published:** 2020-05-09

**Authors:** Noura Al-Zamel, Suleiman Al-Sabah, Yunus Luqmani, Lobna Adi, Siby Chacko, Tom Dario Schneider, Cornelius Krasel

**Affiliations:** 1 Department of Pharmacology & Toxicology, Faculty of Medicine, Kuwait University, PO Box 24923, 13110 Safat, Kuwait; n.alzamel@HSC.EDU.KW (N.A.-Z.); lobna@hsc.edu.kw (L.A.); siby@hsc.edu.kw (S.C.); 2 Department of Pharmaceutical Chemistry, Faculty of Pharmacy, Kuwait University, PO Box 24923, 13110 Safat, Kuwait; yunus@hsc.edu.kw; 3 Institute of Forensic Medicine, Department of Forensic Pharmacology and Toxicology, University of Zurich, 190/52 CH-8057 Zurich, Switzerland; tom.schneider@irm.uzh.ch; 4 School of Pharmacy, Institute for Pharmacology and Toxicology, The Philipps University of Marburg, Karl-von-Frisch-Straße, 135033 Marburg, Germany; cornelius.krasel@staff.uni-marburg.de

The author wishes to make the following correction to this paper [1]. Due to mislabeling, replace: 
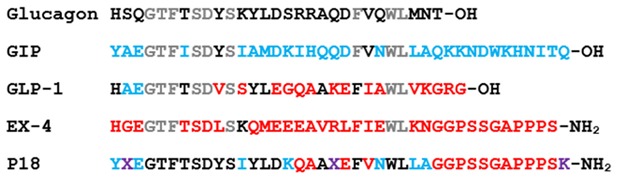
 with 
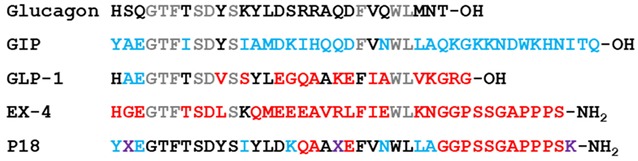


There is an error in the peptide sequence of GIP in Figure 1. Two amino acid residues are missing. The correct sequence for GIP is: YAEGTFISDYSIAMDKIHQQDFVNWLLAQKGKKNDWKHNITQ. The authors would like to apologize for any inconvenience caused to the readers by these changes.
